# Performance and comparability of laboratory methods for measuring ferritin concentrations in human serum or plasma: A systematic review and meta-analysis

**DOI:** 10.1371/journal.pone.0196576

**Published:** 2018-05-03

**Authors:** Maria N. Garcia-Casal, Juan P. Peña-Rosas, Eloisa Urrechaga, Jesus F. Escanero, Junsheng Huo, Ricardo X. Martinez, Lucero Lopez-Perez

**Affiliations:** 1 Evidence and Programme Guidance, Department of Nutrition for Health and Development, World Health Organization, Geneva, Switzerland; 2 Hospital Galdakao Usansolo, Galdakao, Spain; 3 Department of Pharmacology and Physiology, Faculty of Medicine, University of Zaragoza, Zaragoza, Spain; 4 Department of Food Science and Technology, Institute of Nutrition and Food Safety, Beijing, China; Holbæk Hospital, DENMARK

## Abstract

**Background:**

Different laboratory methods are used to quantify ferritin concentrations as a marker of iron status. A systematic review was undertaken to assess the accuracy and comparability of the most used methods for ferritin detection.

**Methods and findings:**

National and regional databases were searched for prospective, retrospective, sectional, longitudinal and case-control studies containing the characteristics and performance of at least one method for serum/plasma ferritin determinations in humans published to date. The analysis included the comparison between at least 2 methods detailing: sensitivity, precision, accuracy, predictive values, inter-methods adjustment, and use of international reference materials. Pooled method performance was analyzed for each method and across methods.

**Outcomes:**

Search strategy identified 11893 records. After de-duplication and screening 252 studies were assessed, including 187 studies in the qualitative analysis and 148 in the meta-analysis. The most used methods included radiometric, nonradiometric and agglutination assays. The overall within-run imprecision for the most reported ferritin methods was 6.2±3.4% (CI 5.69–6.70%; n = 171), between-run imprecision 8.9±8.7% (CI 7.44–10.35%; n = 136), and recovery rate 95.6% (CI 91.5–99.7%; n = 94). The pooled regression coefficient was 0.985 among all methods analyzed, and 0.984 when comparing nonradiometric and radiometric methods, without statistical differences in ferritin concentration ranging from 2.3 to 1454 μμg/L.

**Conclusion:**

The laboratory methods most used to determine ferritin concentrations have comparable accuracy and performance. Registered in PROSPERO CRD42016036222.

## Introduction

Ferritin is an iron storage protein present in all cells of the organism. A small amount is found in plasma and serum, which is a reflection of iron stores in healthy individuals [1, 2]. A low serum ferritin concentration is usually regarded as an indicator of iron depletion, although the interpretation of normal or high serum ferritin values is challenging in the presence of acute or chronic inflammatory processes [3, 4], as ferritin is increased in iron overload states and inflammation [5, 6, 7].

Since ferritin concentration is widely used as marker of iron stores and status, it is important to determine if all methods commonly used to assess ferritin concentrations are capable of detecting and discriminating all possible iron statuses (deficiency, repletion, and overload), and to assess the comparability of methods across measurement systems, The World Health Organization (WHO) Expert Committee on Biological Standardization has established international reference materials to develop tests or to evaluate inter-laboratories performance. These reference materials for ferritin have been developed for calibrating working/secondary standards in the routine ongoing assays performed in laboratories and also for evaluating and standardizing new assays for ferritin quantification. At least three international reference materials have been developed: 1^st^ (liver), 2^nd^ (spleen) and 3^rd^ (recombinant) [8, 9, 10].

WHO is updating its global guidelines on the use of serum and plasma ferritin thresholds for diagnosis of iron deficiency and risk of iron overload [11, 12]. While currently WHO recognizes that ferritin is typically assessed in serum or plasma with enzyme immunoassays after venous blood collection, there is no specific recommendation on variability among analytical methods and commutability [13].

The aim of the present review was to analyze the performance and comparability of the most common laboratory methods used for serum or plasma ferritin concentration determinations to detect iron deficiency, repletion or overload in order to inform decisions on the need for adjustments in the interpretation of serum or plasma ferritin using different methods. This was achieved by determining the sensitivity, specificity and predictive value between ferritin methods; assessing the variability of serum or plasma ferritin concentrations using different laboratory methods of detection; and reviewing the use of international standard materials of ferritin for calibration purposes and in global public health surveillance.

## Methods

### Search strategy and selection criteria

A search strategy and structured search was performed and updated in March 27, 2017. The search strategy for MEDLINE is shown in **Table 1**. The strategy was adapted to the following international and regional databases: Cochrane Central Register of Controlled Trials, MEDLINE, Embase, CINAHL, Science Citation Index, Conference Proceedings Citation Index-Science, BIOSIS Previews, ARIF Reviews Database, PROSPERO, Database of Abstracts of Reviews of Effects (DARE), Cochrane Database of Systematic Review, IBECS, Scielo, Global Index Medicus–AFRO, EMRO, LILACS, PAHO, WPRO, IMSEAR, IndMED, and Proquest Dissertations and Theses. There were no language or publication date restrictions.

**Table 1:** Ovid MEDLINE search strategy.

1 exp Ferritins/ (17454) Note: Lines 1 to 4 are the terms describing the target "ferritin"
2 ferritin*.mp. (25116)
3 isoferritin*.mp. (328)
4 or/1-3 (25330)
5 exp Enzyme-Linked Immunosorbent Assay/ (131202) Note: Lines 5 to 30 are the terms for capturing the five index tests
6 Immunoassay/ (23984)
7 immunoassay*.tw. (51865)
8 assay*.tw. (758672)
9 Radioimmunoassay/ (64971)
10 radioimmunoassay.tw. (47406)
11 RIA.tw. (17348)
12 "Nephelometry and Turbidimetry"/ (6562)
13 turbidmetr*.tw. (8)
14 Nephelometr*.tw. (2416)
15 Luminescence/ (7001)
16 Luminescent Measurements/ (24362)
17 Luminescen*.tw. (12893)
18 chemiluminescen*.tw. (17107)
19 spectrometr*.tw. (160295)
20 (ELISA or CLIA or ECLIA).tw. (115556)
21 Colorimetry/ (18069)
22 colorimetr*.tw. (84)
23 Radiometry/ (28422)
24 radiometr*.tw. (3627)
25 quantum dot*.tw. (6160)
26 micro array*.tw. (790)
27 or/5-26 (1199654)
28 4 and 27 (2646)
29 Ferritins/du [Diagnostic Use] (283)
30 28 or 29 (2922)
31 exp animals/ not humans/ (4109551)
32 30 not 31 (2297)
33 Ferritin bound iron

The protocol “Accuracy and comparability of methods for measuring ferritin concentration to determine iron deficiency, repletion and overload” was registered in PROSPERO International prospective register of systematic reviews (9 March, 2016 under the number CRD42016036222 (https://www.crd.york.ac.uk/PROSPERO/display_record.asp?ID=CRD42016036222)

Prospective, retrospective, sectional, longitudinal and case-control studies containing the characteristics and performance of at least one method for serum or plasma ferritin determinations for human use were included, as well as studies evaluating the performance of two or more ferritin methods comparing the same human samples or using international reference materials. We also considered studies comparing homemade, commercial, automated or single equipment laboratory methods to determine: method type and sub-type, calibration to international reference material, limit of detection, reference interval, linearity and interferences.

Included studies contained data on participants of any age or gender that reported human serum or plasma ferritin concentrations in apparently healthy, iron deficient and/or iron overloaded populations. Studies from infection/inflammation settings, malaria areas, disaster or emergency areas, bacterial versus viral infections, acute versus chronic conditions, and data from diabetic, obese, overweight and/or, insulin-resistant individuals, were also included. Iron deficiency, iron repletion, and iron overload was defined by the trialists. If not specified, WHO cut-offs [13] were used.

The studies containing data using international reference materials (especially either of the WHO reference materials from spleen, liver or recombinant) to calibrate commercial assays, automated equipment or used in the routine ongoing assays performed in laboratories, were extracted, sub grouped and recorded in order to register the frequency of use in commercial assays and automated equipment developments, and in routine laboratory assays and surveys globally.

The primary outcomes were characteristics and performance indicators of ferritin methods: type (radiometric, nonradiometric, agglutination), sub-type (EIA, turbidimetric, chemiluminescent, RIA, IRMA), origin of the assay (homemade, commercial), detection equipment (single apparatus, automated multiple-analytes detection equipment), sensitivity, specificity, precision, limit of detection, recovery rate, within-run and between-run imprecision. Data comparing at least two methods for ferritin determination and extracted correlation coefficient, coefficient of agreement or Bland-Altman Plot or regression coefficients were also recorded. The use of international reference materials was also summarized including the origin, use for calibration while developing the assay or use on a routine basis.

### Data collection and analysis

The data retrieved in each search was screened independently by two authors to assess eligibility. Selected records were independently extracted using data extraction forms tested and approved by all authors in order to enhance consistency amongst reviewers. Disagreements at any stage of the eligibility assessment process were resolved through discussion and consultation with a third author.

The information collected included: general information, sample size, baseline characteristics (iron status, age, sex, race, presence and severity of infection/inflammation), ferritin detection method(s) used, cutoff points used to define iron deficiency, repletion or overload, methodological details on ferritin quantification (method(s) used for ferritin determination(s) and the characteristics of the test already mentioned as outcomes.

**Fig 1:**
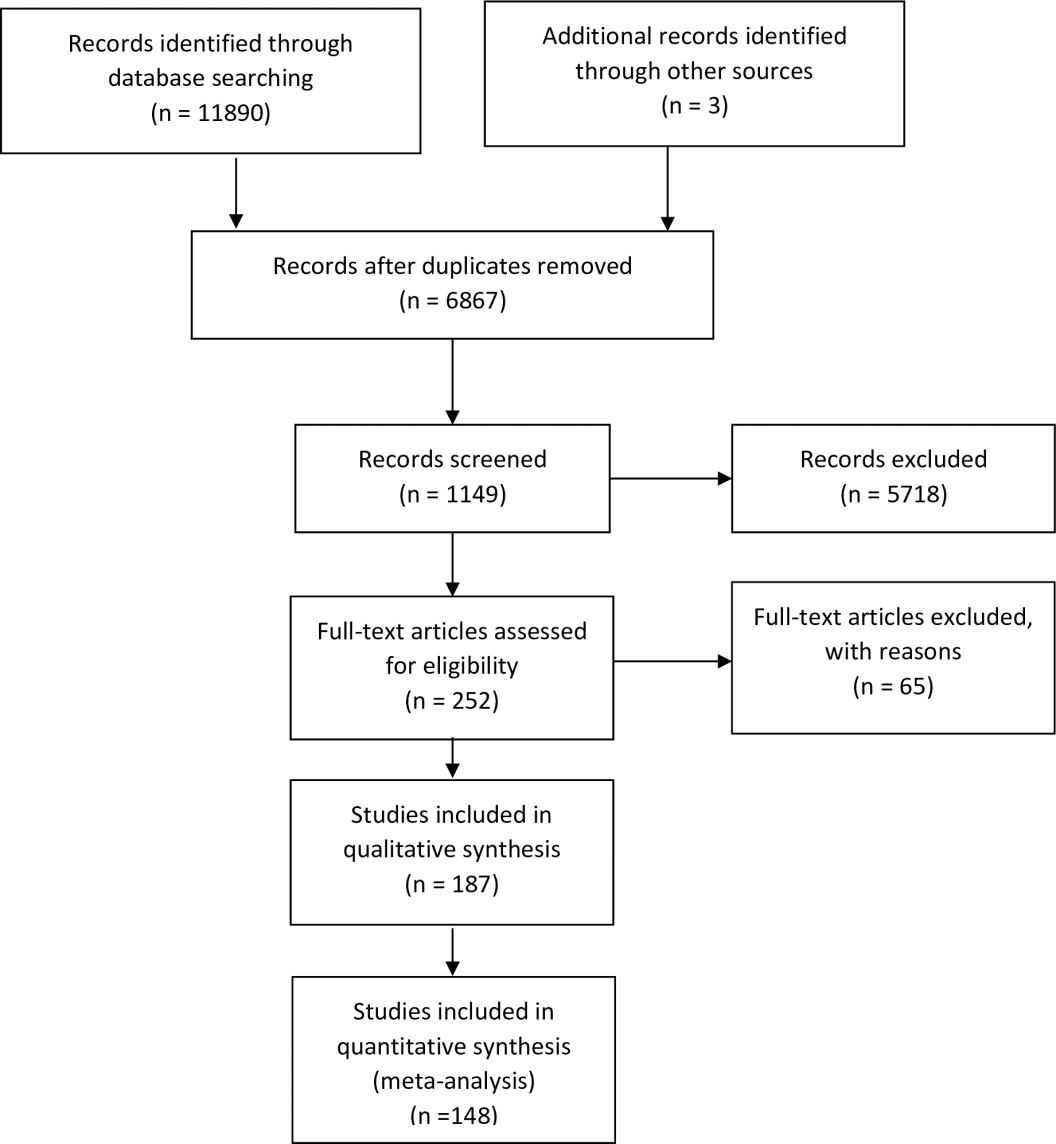
PRISMA flow-chart of study selection.

We included a PRISMA (preferred reporting items for systematic reviews and meta-analyses) flow-chart of study selection (**Fig 1**) [14].

The variability of ferritin determinations was assessed by: 1. intrinsic indicators, commonly used to assess the characteristics of a particular ferritin method (e.g. precision and accuracy); and 2. comparative indicators between two ferritin methods, such as correlation coefficient.

The intrinsic indicators chosen to characterize the different ferritin methods were within-run imprecision, between-run imprecision, recovery rate, minimal level of detection (μg/L) and assay linearity.

The comparative indicators between different ferritin methods included correlation coefficient (ρ), noise or disturbance term of the regression equation (intercept b) and slope of the regression equation (m). To assess comparability between methods we compute statistics of the corresponding linear regression equation parameters and fixed effects meta-analysis of the correlation. To estimate inter-methods adjustments, a search for laboratory assessment data was performed. The search included laboratory performance data or reports on internet and also directly contacted some laboratories to ask for the possibility of data sharing Bland-Altman plots and/or statistics were extracted if present in the selected studies.

The correlation coefficient between methods was performed by standard correlation fixed-effects meta-analysis, because the variance depends strongly on the correlation, the last is converted to the Fisher’s z scale, and all analyses were performed using the transformed values. All statistical analysis was performed using Stata (https://www.jmp.com/en_gb/), MetaXL (http://www.epigear.com/) and CMA (http://www.comprehensive.com) softwares.

Originally, covariate analysis, development of Summary Receiver Operating Curves (SROC) and accuracy estimates were planned for the following groups: a) test type or sub-type: radiometric, nonradiometric, agglutination, other; b) origin of the assay: homemade versus commercial c) detection equipment/system: single apparatus versus automated detection equipment; d) assay performance: variability and reproducibility; e) sample matrix: serum, plasma or erythrocytes; f) age group: infants (less than one year of age), children (two to 11.9 years of age), adolescents (12 to 18.9 years of age), adults (19 years of age or older); g) gender; h) vulnerability to iron deficiency: infants, women of reproductive age, pregnancy; i) body mass index (BMI); j) infection/inflammation: malaria area, emergency settings, groups of patients with inflammatory conditions; k) capability of both-ends detection: iron deficiency, sufficiency and overload; l) correlation of methods by publication year; m) calibration to international reference materials. The analyses were performed for the methods and subgroups with enough available data and included: test type or sub-type (radiometric, nonradiometric, agglutination); origin of the assay (homemade versus commercial); detection equipment/system (single apparatus versus automated detection equipment); assay performance (variability and reproducibility); limit of detection; calibration to international reference materials.

### External laboratory quality assessment data

Data on ferritin measurement precision from laboratory quality assessment programs was searched and extracted to compare reported between-laboratory means and coefficients of variation.

### Use of international reference materials

For assessing the use of international reference materials, we developed comparative tables for the most common used methods sub classified as homemade, commercial and automated.

For all analysis, selection of indicators was dictated by data availability and consistency. At least three studies reporting on a specific indicator detected by the same method type or sub-type were required to perform meta-analysis estimates. Differences between covariates were assessed by visual inspection; non-overlapping confidence intervals (CIs) suggested a statistically significant difference in treatment effect between the subsets.

## Results

The PRISMA flow chart of study selection is presented in **Fig 1**. Eleven thousand eight hundred ninety three records were obtained from the search strategy depicted in **Table 1**. From 252 records extracted, 187 studies were included in the qualitative analysis [8,10,15–199] and 148 in the meta-analysis [8,10,15,17,18,22,23,26,28,30–32,34–56,58–64,66–68,72,73,75,77–80,82–85,87–92,94–98,100–102,106–109,111–116,120–137,139–143,145,146,148–151,153–157,159–164,166–175,177,179–181,183,184,186–193,195–199]. Many of the studies included in the meta-analysis provided more than one entry for analysis (some of them compare and analyze up to 11 methods for ferritin determinations [95,112,139,145,193], resulting in some analysis including up to 233 entries. In order to summarize the information from each indicator, pooled estimates were performed for recovery rates and regression coefficients. The characteristics of the studies included in the meta-analysis are presented in **S1 Table,** as supplementary material.

### Within-run imprecision for method type or sub-type, origin of the assay and detection equipment

For analysis of within-run imprecisions, the methods more commonly used were 94 nonradiometric immunoassays (including 25 enzyme linked immuno sorbent assay (ELISA) and 25 chemiluminescent), 59 radiometric (27 radioimmunoassays (RIA) and 32 immunoradiometric assay (IRMA)), and 17 based on agglutination (including 13 turbidimetric and 3 nephelometric).

The overall mean within-run imprecision for the most reported ferritin methods was 6.2±3.4% (CI 5.69–6.70%; n = 171). The method with the highest within-run imprecision was the enzyme immunoassay with fluorometric detection (8.3±6.3%; CI 3.66–12.95%; n = 7), and the lowest variation was obtained with nephelometric methods (2.3± 0.4%; CI 1.84–2.70%; n = 3). Differences were not significant between methods except when comparing with nephelometry, although the sample size was only 3 data entries (**Table 2).**

**Table 2:** Within-run imprecision of the methods commonly used for serum or plasma ferritin determinations grouped by method type and sub-type.

Method type and sub-type^1^	Number of data entries	Mean CV^3^ (%)	STD^2^ (%)	95% confidence interval (%)	
				Lower limit	Upper limit
**Labelled nonradiometric**					
EIA	31	5.8	2.5	4.43	6.98
Fluorimetric	7	8.3	6.3	3.66	12.95
ELISA	25	7.5	3.3	6.24	8.80
Chemiluminescent	25	4.8	2.3	3.88	5.72
MEIA	5	4.3	1.8	2.64	5.87
RPIA	1	4.9			
**Labelled radiometric**					
RIA	27	7.5	4.0	5.97	8.98
IRMA	32	6.7	3.0	5.65	7.74
**Agglutination**					
Turbidimetric	13	4.3	3.1	2.57	2.94
Nephelometric	3	2.3	0.4	1.84	2.70
LPIA	1	3.1			
**Other**	1	5.4			
**All**	**171**	**6.2**	**3.4**	**5.69**	**6.70**

^1^ EIA: Enzyme immunoassay; ELISA: Enzyme linked immunosorbent assay; MEIA: microparticle enzyme immunoassay; RIA: Radioimmune assay; IRMA: Immunoradiometric assay; LPIA Latex photometric immunoassay; RPIA: Radial partition immunoassay.

^2^ Standard deviation.

^3^ Coefficient of variance.

**Table 3** shows the mean within-run imprecision when grouping methods by type as nonradiometric, radiometric and agglutination; by the origin of the assay (homemade, commercial), and by detection equipment (single apparatus, automated detection equipment). As reported before, agglutination methods showed the lowest within-run imprecision (3.8±2.8%; 95% CI 2.5–5.17; n = 17), and radiometric methods the highest (7.1±3.5%; 95% CI 6.16–7.94 n = 59). Commercial assays had lower within-run imprecision s (5.9±3.3%; 95% CI 5.26–6.45; n = 115) than homemade kits (6.9±3.5%; 95% CI 5.96–7.82; n = 56), although differences were not statistically significant. Likewise, automated multiple-analytes detection equipment had lower within-run imprecision s (5.3±2.7%; 95% CI 4.67–5.86; n = 78), compared to single laboratory apparatus (7.0±3.7%; 95% CI 6.22–7.72; n = 93), but differences were not significant.

**Table 3:** Within-run imprecision of the methods commonly used for serum or plasma ferritin determinations grouped by method type, by origin of the assay and by detection equipment.

	Number of data entries	Mean CV^3^ (%)	STD^1^ (%)	95% confidence interval (%)	
				Lower limit	Upper limit
**Method type**					
Labelled nonradiometric	94	6.1	3.2	5.43	6.74
Labelled radiometric	59	7.1	3.5	6.16	7.94
Agglutination	17	3.8	2.8	2.50	5.17
Other	1	5.4			
**All**	**171**	**6.2**	**3.4**	**5.69**	**6.70**
**Origin of assay**					
Commercial	115	5.9	3.3	5.26	6.45
Homemade	56	6.9	3.5	5.96	7.82
**Detection equipment**					
Automated	78	5.3	2.7	4.67	5.86
Single app^2^	93	7.0	3.7	6.22	7.72

^1^ Standard deviation.

^2^ Single apparatus.

^3^ Coefficient of variance.

### Between-run imprecision for detection method, origin of the assay and detection equipment

For analysis of between-run imprecision the methods more commonly used were 75 nonradiometric immunoassays (including 25 ELISA and 17 chemiluminescent), 47 radiometric (22 RIA and 25 IRMA), and 14 based on agglutination (including 10 turbidimetric and 3 nephelometric).

The overall mean between-run imprecision for the most reported ferritin methods was 8.9±8.7% (95% CI 7.44–10.35; n = 136). The method with the highest between-run imprecision was the nonradiometric immunoassay with fluorometric detection (24.8±41.5%; 95% CI 0.00–61.21; n = 5), and the lowest variation was obtained with nephelometric methods (4.1± 0.1%; 95% CI 3.99–4.21; n = 3). Variations from chemiluminescent methods (6.9± 2.6%; 95% CI 5.70–8.14; n = 17) were not different from light passage ones. Differences were not significant between methods as the confidence intervals with the other assays have subtle intersections **(Table 4).**

**Table 4:** Between-run imprecision of the methods commonly used for serum or plasma ferritin determinations grouped by method type and sub-type.

Method type and sub-type^1^	Number of data entries	Mean CV^2^ (%)	STD^3^ (%)	95% confidence interval (%)	
				Lower limit	Upper limit
**Labelled nonradiometric**					
EIA	24	8.1	3.7	7.21	10.06
Fluorimetric	5	24.8	41.5	0.00	61.21
ELISA	25	10.1	3.6	8.68	11.52
Chemiluminescent	17	6.9	2.6	5. 70	8.14
MEIA	4	5.2	0.4	4.76	5.59
**Labelled radiometric**					
RIA	22	10.0	4.3	8.15	11.76
IRMA	25	8.7	3.3	7.45	10.00
**Agglutination**					
Turbidimetric	10	4.3	3.0	2.41	6.10
Nephelometric	3	4.1	0.1	3.99	4.21
LPIA	1	2.8			
**All**	**136**	**8.9**	**8.7**	**7.44**	**10.35**

^1^ EIA: Enzyme immunoassay; ELISA: Enzyme linked immunosorbent assay; MEIA: microparticle enzyme immunoassay; RIA: Radioimmune assay; IRMA: Immunoradiometric assay; LPIA Latex photometric immunoassay; RPIA: Radial partition immunoassay.

^2^ Coefficient of variance.

^3^ Standard deviation.

When methods were grouped by type, agglutination methods showed the lowest between-run imprecision (4.1±2.5%; 95% CI 2.81–5.43; n = 14), and nonradiometric methods the highest (9.5±11.0%; 95% CI 7.03–12.03; n = 75), without differences with radiometric ones (9.3±3.8%; 95% CI 8.21–10.39; n = 47). Between-run imprecisions from commercial assays (9.1±10.1%; 95% CI 7.03–11.10; n = 95) were not different than from homemade assays (8.5±3.4%; 95% CI 7.45–9.52; n = 41). Automated multiple-analytes detection equipment had between-run imprecisions (8.9±12.2%; 95% CI 5.89–11.95; n = 62), not statistically different from single laboratory apparatus (8.9±3.8%; 95% CI 7.99–9.74; n = 74) **(Table 5).**

**Table 5:** Between-run imprecision of the methods commonly used for serum or plasma ferritin determinations grouped by method type, by origin of the assay and by detection equipment.

	Number of data entries	Mean CV^3^ (%)	STD^1^ (%)	95% confidence interval (%)	
				Lower limit	Upper limit
**Method type**					
Labelled nonradiometric	75	9.5	11.0	7.03	12.03
Labelled radiometric	47	9.3	3.8	8.21	10.39
Agglutination	14	4.1	2.5	2.81	5.43
**All**	**136**	**8.9**	**8.7**	**7.44**	**10.35**
**Origin of assay**					
Commercial	95	9.1	10.1	7.03	11.10
Homemade	41	8.5	3.4	7.45	9.52
**Detection equipment**					
Automated	62	8.9	12.2	5.89	11.95
Single app^2^	74	8.9	3.8	7.99	9.74

^1^ Standard deviation.

^2^ Single apparatus.

^3^ Coefficient of variance.

### Recovery rate for method type and sub-type, origin of the assay and detection equipment

The pooled recovery rate for all assays evaluated (n = 94) was 95.6% (95% CI 91.5–99.7). When grouped by method type, agglutination methods showed the lower recoveries (88.1%; 95% CI 80.7–95.4; n = 18) compared to nonradiometric immunoassays that included 12 ELISA and 12 chemiluminescent methods (97.7%; 95% CI 92.1–103.29; n = 54) or to radioactive methods (103.8%; 95% CI 93.1–114.3; n = 22). There was no difference in recoveries between commercial and homemade assays (94.8%; 95%CI 89.9–99.7; n = 61 vs. 97.6%; 95%CI 89.9–105.2; n = 33, respectively), or between automated multiple-analytes detection equipment and single laboratory apparatus (94.8%; 95% CI 89.7–99.7; n = 54 vs. 97.4%; 95% CI 90.3–104.5; n = 40, respectively).

The recovery rates did not changed significantly when data was analyzed by the ferritin concentration added. In studies spiking ferritin up to 100 μg/L (n = 32), recovery was 92.2% (95% CI 85.0–99.3); 97.0% (95% CI 89.7–104.2) for concentrations up to 350 μg/L (n = 30), and 98.2% (95% CI 83.0–109.5) for concentrations up to 1000 μg/L (n = 25).

### Limit of detection

The lowest average levels of detection were found in nonradiometric methods (2.3±4.5μg/L; n = 72) and agglutination methods (2.6±2.5μg/L; n = 14), compared to radiometric methods (7.3± 12.5μg/L; n = 47). There was no significant difference in average lowest levels of detection between commercial and homemade kits (4.2± 9.4μg/L; n = 96 vs. 3.8± 5.5μg/L; n = 38, respectively), or between automated multiple-analytes detection equipment and single laboratory apparatus (2.6± 3.5μg/L; n = 63 vs. 5.5± 10.9μg/L; n = 71, respectively).

The highest average level of detection was found in agglutination methods 1454± 1517μg/L; n = 14), compared to nonradiometric (1171± 1145μg/L; n = 72), and radiometric methods (808±1068μg/L; n = 47). There was no significant difference in average highest levels of detection between commercial and homemade assays (1070± 1025μg/L; n = 96 vs. 1053± 1494μg/L; n = 38, respectively), or between automated multiple-analytes detection equipment and single laboratory apparatus (1151± 1068μg/L; n = 63 vs. 989± 1258μg/L; n = 71, respectively).

### Assay linearity for method type, origin of the assay and detection equipment

The most reported maximal ferritin concentrations that maintained assay linearity were 500 and 1000μg/L (11 out of 56 assays). The highest concentration reported to maintain linearity of the curve was 6000μg/L. The highest concentrations that maintained linearity (2000–6000μg/L) were no related to a particular method or equipment; they were found with radiometric, nonradiometric or homemade assays as well as with agglutination or commercial assays. Likewise, the lowest linearity concentration reported was 100μg/L, and it was not related to origin of the assay (commercial or homemade), but the 3 studies that reported such linearity threshold, were nonradiometric.

### Correlation between methods

**Table 6** shows a correlation of 0.981 between all methods analyzed. Correlation coefficient was 0.984 when comparing nonradiometric and radiometric methods.

**Table 6:** Correlation coefficient fixed effects meta-analysis for all methods and comparison between nonradiometric and radiometric methods (pooled estimate computed using standard Fisher’s Z transform).

Type of Assay	Correlation coefficient Estimate	95% confidence interval		Number of data entries
		Lower limit	Upper limit	
Nonradiometric vs nonradiometric	0.989	0.988	0.989	107
Nonradiometric vs radiometric	0.984	0.983	0.985	74
Radiometric vs radiometric	0.878	0.869	0.886	51
**All**	**0.981**	**0.980**	**0.981**	**233**

The correlation coefficient was further analyzed at various serum ferritin concentrations. There were no statistically significant differences in correlation coefficients between methods when analyzing samples containing up to 300 μg/L (0.94± 0.06; 95% CI 0.928–0.961; n = 44), up to 800 μg/L (0.99± 0.01; 95% CI 0.984–0.993; n = 42), up to 1500 μg/L (0.98± 0.02; CI 0.972–0.985; n = 45) or even higher concentrations (0.98± 0.03; CI 0.961–0.989; n = 23).

### Intercept (b) and slope (m) of the regression equation

These comparative indicators were found in 85% of the selected articles (115/136), with 218 data entries. As seen in **Table 7**, intercept values were far from 0 although not significantly different from it for the comparisons between nonradiometric methods or between no radiometric and radiometric methods, showing a high variability with standard deviations between 8.85 and 20.83 μg/L. For the three comparisons, slopes were close to 1.

**Table 7:** Mean and variance of the regression intercept and slope between nonradiometric and radiometric assays.

Type of Assay	Mean intercept ±SD	95% confidence interval		Number of data entries
		Lower limit	Upper limit	
**Intercept**	μg/L			
Nonradiometric vs nonradiometric	2.72±14.14	-0.070	5.500	99
Nonradiometric vs radiometric	-4.14±20.83	-8.919	0.640	73
Radiometric vs radiometric	3.99±8.85	1.487	6.496	48
**Slope**				
Nonradiometric vs nonradiometric	0.93±0.57	0.818	1.041	99
Nonradiometric vs radiometric	0.95±0.32	0.875	1.023	73
Radiometric vs radiometric	1.11±0.45	0.981	1.236	48

### Agreement between laboratory methods

It is important to highlight that the use of the correlation, intercept, and slope indicators in our statistical analysis, only indicated the strength of the relationship or the linearity between compared methods, not the agreement between them. The most used agreement indicator Bland-Altman plots [200] and/or statistics, was only reported in seven studies [62, 68, 79, 115, 142, 162, 198] (**S2 Table,** supplementary material).

A recent study [115] found poor agreement (bias from -11.5 to 44 μg/L and σ higher than 25 μg/L) between ELISA vs microparticle enzyme immunoassay (MEIA) or chemiluminiscence. Previously, two other studies [68, 162] found moderate agreement (bias from -8 to 3.7 μg/L and σ from 8.5 to 12 μg/L) between ELISA versus chemiluminescence and radiometric methods, respectively. Zhang 2015 [198], and Dipalo 2016 [62] found moderate to poor agreement between chemiluminiscence (bias from 34 to 60 μg/L and σ from 12 to 71 μg/L), while Molinario 2015 [142] and Gomez 2000 [79] found good agreement between agglutination and chemiluminescent methods (bias from -6 to -7.9 μg/L and σ from 1.5 to 8 μg/L).

In these seven studies, the authors stated that when ferritin concentrations were below a certain threshold (75 μg/L for three of the studies) [68, 79, 162], the agreement improved considerably. These results cannot be generalized because of the low number of studies reporting this indicator, although illustrate the limitations in interchangeability, especially above certain ferritin concentrations.

### Laboratory assessment data

Data on ferritin measurement precision were obtained from four laboratory quality assessment programs, two reporting on between-laboratory means and coefficients of variation [201, 202], other [203] on reference range data (lowest limits for ferritin range) and the forth on commutability and deviation from the mean [126]. Based on the highest number of participating laboratories, a report from the XXI Laboratory Quality Assessment Program of the Spanish Society of Clinical Biochemistry and Molecular Pathology in 2014 was selected for analysis of ferritin results [202].

One hundred and eighty eight laboratories participated in this round, and most of them used automated equipment. The methods used for detection in these automated equipment were immunoenzymatic with different antigen-antibody pairs and detection systems (chemiluminiscence, agglutination). The confidence intervals and coefficient of variation calculated from this report are presented in **S3 Table** (supplementary material), showing a between-laboratory coefficient of variation from 7.52% to 13.10%, which is in agreement with our findings of a mean CV of 8.5% for EIA and 6.8% for chemiluminescence.

### Use of international reference materials

As shown in **S1 Table** (supplementary material), some of the studies report calibrating the assays to WHO or other international reference materials, although calibration data is not presented in the articles [8,10,18,36,38,45,54,62,78,79,85,95,112,115, 116, 126,131,136,137,139,140,142,148,155,156,158,162,164,168,171,187,190]. There were no studies reporting the use of these materials on a laboratory routine basis. Only one study reported results from a serum pool ‘spiked’ with either the 1^st^ and 2^nd^ international standard for ferritin. Those sera were measured by 52 laboratories using five automated methods and the recovery of the target values was calculated. The recovery of the first international reference materials by three of the methods was between 104 and 129%; and to the second reference material was between 99 and 125%. Authors recommend manufacturers to calibrate their methods against the 3^rd^ international standard (recombinant), and to periodically assess their methods relative to this standard as a means of avoiding assay drift over time [36].

## Discussion

Measurement of iron status in the general population is important to determine the prevalence and distribution of iron deficiency and overload, and thus to decide appropriate interventions, and to monitor and evaluate the impact and safety of implemented public health programmes.

The selection of a test that reflects real ferritin concentration and has been validated through the use of reference materials has important implications from the perspective of the individual and public health pathway. The comparability of results from patients for differential diagnosis of iron deficiency or risk for iron overload has important implications for clinical decisions and also the appropriate use of resources. It is important to determine whether a therapeutic decision and treatment is having the expected effect and is not causing harm, regardless of the laboratory method used. Likewise, in the planning and evaluation of public health interventions it is important to determine the iron status of the targeted population as well as the impact of a nutrition-specific or nutrition-sensitive intervention. If different laboratory methods give different results, this may lead to misinterpretation of the effects and lead to incorrect public health decisions. Additionally, it should be possible to compare data from different surveys performed years apart and performed by different methods.

There are various methods to determine serum, plasma or erythrocyte ferritin concentration. The vast majority of them are based in antigen-antibody reactions that are detected by different methods. The detection could be by radioactive counting or by color development using different enzyme-substrate pairs to measure color development at visible range. Also, light scattering or turbidimetric measurements have been developed as well as fluorescence emission. The first reported methods for ferritin determination were radioactive, using radioisotopes to label the antigen (IRMA) or the antibody (RIA) [16, 61]. Common colorimetric methods include peroxidase or glycosidase based color development [177, 204, 205]. Methods based in agglutination and light passage could be turbidimetric or nephelometric [35, 206]. More recently, quantum dots, plasma-mass spectrometry, and micro array based technologies have been developed [207, 208].

Another milestone in the development of methods for ferritin determination was automatization. Radioactive methods started as home made in a few laboratories, followed in few years by colorimetric tests. Both methodologies were improved and commercialized, making them widely available. More recently, automated equipment that allow the determination of various metabolites, including ferritin, have been developed [34, 157, 209]. The detection method for these equipment vary, but is mainly based on turbidimetry and chemiluminescence.

The evolution of routine medical laboratories is reflected in the different techniques applied for ferritin determination: the principle of the method used for detection has changed, as well as the use of automated equipment. Our results show that the methods used in the included studies and the use of automated or single apparatus equipment for detection, made no difference for ferritin determinations.

This analysis has limitations. It was not possible to identify a gold standard method for ferritin determinations. This precluded the possibility of comparing ferritin methods against a reference method or to develop 2x2 tables. Instead, individual method performance and comparisons between all available methods that contained consistent data were analyzed.

In most of the included studies there were no details on the characteristics of the human serum or plasma used to test or validate ferritin assays, and it was not possible to sub classify studies by iron status (deficiency, repletion or overload), physiological state, age, gender or inflammatory conditions of the samples used to describe or compare ferritin methods. In fact, many valuable studies do not report on the methods used to determine ferritin. A recent systematic review summarized the serum ferritin thresholds recommended by international professional organizations associations worldwide to define iron deficiency in different population groups [210]. There was no mention on the preferred or recommended laboratory methods or the commutability between them to interpret the results.

Although it was not possible to perform comparisons or meta-analysis, available studies report the use of WHO or other international reference materials while developing and calibrating an assay, but also to improve comparability between methods and assays components [10, 36, 87, 88, 96, 139, 140, 181]. Studies reporting on commercial, automated equipment refer the use of international reference materials for calibration while developing an assay [96]. One study on accuracy and precision of seven commercial kits for serum ferritin (3 RIAs and 4 IRMAs), demonstrate that the use of a reference ferritin standard improved the accuracy of serum ferritin determination, but did not eliminate the variability of the determinations particularly for high ferritin concentrations [95].

The main challenges identified with the development of this review were related to the variety of available methods and small practical differences between them, which resulted in a challenge for categorization of methods. Other important issue was the quality of publications, especially for automated and commercial assays. In many cases they were only reports or abstract to scientific meetings, that did not result in a posterior formal, complete publication. The use of a Diagnostic Test Accuracy (DTA) methods approach to develop this review was not possible due to the difficulty to unequivocally identify a gold standard method to perform comparisons.

Regarding statistical analysis, it was not possible to perform meta-analysis of important indicators such as Bland-Altman statistics (mean differences and corresponding variance), repeatability, high dose hook effect and carry over due to the limited amount and quality of data. These limitations also come from the fact that the studies were not trying to compare accuracies of existing methods, but focused on similarities between two methods that were available to them. In fact, more than 98% of the included articles did not study methods agreement or use proper method comparison techniques such as Passing-Bablok regressions [112, 142], Spearman correlations, and concordance methods [62].

The results from this review show that the methods used to determine ferritin are comparable and there is not preferred /recommended laboratory method, although the risk for radioactive contamination and expensive equipment are important drawbacks of RIA and IRMA. For patient follow-up, public health surveys or evaluations of impact of interventions it is recommended that, once selected, the same ferritin method should be used during the different stages of the intervention.

International reference materials (e.g. WHO standard) should be used for calibration of all commercial assays and probably in the regular laboratory practice for periodical assessment of the reagents and equipment of the routine method used, providing that the reference material has been shown to be commutable for a particular assay. It is important that reference materials are commutable so the results from samples are equivalent among all procedures, in order to obtain results traceable to the reference system and without calibration bias among procedures. Laboratories performing ferritin determinations for patient care or for public health assessments should participate in national or regional quality control surveys.

## Supporting information

S1 Table Characteristics of the studies included in meta-analysis. (DOCX)

S2 Table Summary of studies reporting Bland-Altman (B-A) statistics: Mean and standard deviation of the regression intercept and slope between different detection techniques. (DOCX)

S3 Table Laboratory assessment data from a quality control program of the Spanish Society of Clinical Biochemistry and Molecular Pathology (2014). (DOCX)

S1 File PRISMA checklist ferritin methods. (DOC)

S2 File Review ferritin methods PROSPERO registration. (PDF)
