# Supplementary material for: Performance and comparability of laboratory methods for measuring ferritin concentrations in human serum or plasma: A systematic review and meta-analysis
Source: PLoS One. 2018 May 3;13(5):e0196576. doi: 10.1371/journal.pone.0196576 (PMC5933730; doi:10.1371/journal.pone.0196576)
Supplement: S1 Table — (DOCX) [file pone.0196576.s001.docx]

**Supporting information**

S1 Table. Characteristics of the studies included in meta-analysis

| ***Reference ID*** | ***Type of publication*** | ***Year of publication*** | ***Method subtype^1^*** | ***Detection equipment*** | ***Commercial brands*** | ***Use of reference STD material for calibration*** |
| --- | --- | --- | --- | --- | --- | --- |
| Hamwi 02 | Article | 2002 | Nephelometry, Chemiluminiscence | Automatized | Behring (N-Latex) | WHO STD reference material (spleen) |
| Thorpe 97 | Article | 1997 | ELISA | Single apparatus | home-made | WHO STD reference material recombinant |
| Abdul-Ahad 92. | Abstract | 1992 | EIA | Single apparatus | Becton Dickinson (affinity) | Not reported |
| Aguanno 84 | Abstract | 1984 | EIA | Single apparatus | Abbott (Ferrizyme) | Not reported |
| Akbas 14 | Article | 2014 | Chemiluminiscence | Automatized | Beckman Coulter (DxI 800) | WHO STD reference material recombinant |
| Alshawi 83 | Article | 1983 | IRMA | Single apparatus | home-made | Other |
| Alshawi 84 | Article | 1984 | IRMA | Single apparatus | home-made | Other |
| Anaokar 79 | Article | 1979 | EIA | Single apparatus | home-made | Not reported |
| Anderson 81 | Article | 1981 | ELISA | Single apparatus | home-made | Not reported |
| Armenta 85 | Article | 1985 | Fluorimetry | Single apparatus | home-made | Other |
| Assink 83 | Article | 1983 | ELISA, IRMA | Single apparatus | Ramco (Spectro Ferritin), Ramco (Fer-Iron), Behring (Ria-gnost) | Not reported |
| Barlow 88 | Abstract | 1988 | Chemiluminiscence | Automatized | Corning (Magic Lite) | Not reported |
| Bernard 83 | Article | 1983 | Turbidimetry | Automatized | home-made | Not reported |
| Bernard 84 | Article | 1984 | Turbidimetry | Single apparatus | home-made | Not reported |
| Blackmore 08 | Article | 2008 | Chemiluminiscence |  |  | WHO STD reference material (spleen) |
| Blick 88 | Abstract | 1988 | IRMA | Automatized | Biomerieux (VITEK 2 system KinetiCount48) | Not reported |
| Blunden 80 | Article | 1980 | IRMA, RIA | Single apparatus | Roche (Sirius), Ramco (Fer-Iron), Clinical Assays (Gamma Dab), home-made | WHO STD reference material (spleen) |
| Borque 92 | Article | 1992 | Nephelometry | Single apparatus | Behring (BNA) | Not reported |
| Borque 96 | Article | 1996 | Nephelometry | Single apparatus | Behring (BNA) | Not reported |
| Borque 99a | Abstract | 1999 | Nephelometry | Automatized | Beckman (IMMAGE) | Not reported |
| Borque 99b | Article | 1999 | Turbidimetry | Single apparatus | Abbott (Falcor 300) | Not reported |
| Bradley 87 | Abstract | 1987 | IRMA | Single apparatus | Not reported | Not reported |
| Brindle 14 | Article | 2014 | ELISA | Automatized | multiplexed MN assessment tool (MMAT) | Other |
| Brotherton 90 | Article | 1990 | Chemiluminiscence, Fluorimetry, EIA, MEIA | Single apparatus | Amersham (Amerlite), LKB (Delphia), Walker (Synelisa), Ramco (Spectro-MT), Abbott (IMx), Flow (Monoscan) | WHO STD reference material (liver) |
| Camara 92 | Article | 1992 | ELISA, fluorimetry, RIA | Automatized | Boehringer Mannheim (ES-300) | Not reported |
| Camilo 14 | Abstract | 2014 | Chemiluminiscence | Automatized | Advia Centaur® XP Siemens | Not reported |
| Chida 85 | Article | 1985 | EIA | Single apparatus | home-made | Not reported |
| Chou 89 | Abstract | 1989 | MEIA | Automatized | Abbott (AxSYM) | Not reported |
| Christopher 90 | Abstract | 1990 | Chemiluminiscence | Automatized | Corning (Magic Lite) | Not reported |
| Cloete 02 | Article | 2002 | EIA | Automatized | Bayer (Technicon Immuno I) | Not reported |
| Conradie 80 | Article | 1980 | ELISA | Single apparatus | home-made | Not reported |
| Cozzi 89 | Article | 1989 | ELISA | Single apparatus | home-made | Not reported |
| Cragle 86 | Abstract | 1986 | EIA | Automatized | Beckman Coulter (epsilon) | WHO STD reference material (liver) |
| Datta 11 | Abstract | 2011 | Turbidimetry | Automatized | ADVIA Chemistry systems Siemens | Other |
| Datta 92 | Abstract | 1992 | Chemiluminiscence | Automatized | Siemens (Immulite 1) | Not reported |
| DCosta 93 | Article | 1993 | ELISA | Automatized | Boehringer Mannheim (ES-300) | Not reported |
| Dempster 79 | Abstract | 1979 | IRMA | Single apparatus | home-made | Not reported |
| Denend 86 | Abstract | 1986 | IRMA | Single apparatus | Bio-Rad (Quantimune) | Other |
| Deppe 78 | Article | 1978 | IRMA | Single apparatus | home-made | Other |
| Dipalo 16 | Article | 2016 | Chemiluminiscence | Automatized | Beckman Coulter DxI 800 and Maglumi 2000 | WHO STD reference material recombinant |
| Donnell 80 | Abstract | 1980 | RIA |  |  | Not reported |
| Dunn 81 | Article | 1981 | IRMA | Automatized | Ramco (Fer-Iron), Corning (Immuno pHase Fer), Ventrex (Ventre/Sep) | Not reported |
| Ellisor 86 | Abstract | 1986 | RPIA | Automatized | Dade (stratus) | Not reported |
| Englebienne 00 | Article | 2000 | Turbidimetry | Automatized | Roche (Cobas-Mira) | Not reported |
| Erhardt 04 | Article | 2004 | ELISA, IRMA | Single apparatus | home-made | Not reported |
| Fortier 78 | Abstract | 1978 | ELISA | Automatized | New England (EIA-01-Fe) | Not reported |
| Fortier 79 | Article | 1979 | ELISA | Automatized | New England (EIA-01-Fe) | Not reported |
| Gaida 92 | Abstract | 1992 | EIA | Single apparatus | home-made | Not reported |
| Ghielmi 82 | Article | 1982 | RIA, IRMA, ELISA | Single apparatus | commercial, home-made | Not reported |
| Goldie 78 | Article | 1978 | RIA | Single apparatus | Bristol | WHO STD reference material (spleen) |
| Gomez 00 | Article | 2000 | Turbidimetry, chemiluminiscence | Automatized | Instrumentation Laboratories (ILab 900 analyzer) | WHO STD reference material recombinant |
| Goodnow 86 | Abstract | 1986 | RPIA | Automatized | Dade (stratus) | Not reported |
| Guerra 98 | Abstract | 1998 | Nephelometry | Automatized | Behring (N-Latex) | Not reported |
| Guilleux 81 | Article | 1981 | RIA | Single apparatus | home-made | Other |
| Haden 87 | Abstract | 1987 | EIA | Automatized | Beckman Coulter (epsilon) | Not reported |
| Hallberg 93 | Article | 1993 | RIA | Single apparatus | Diagnostic Products (RIA) | WHO STD reference material (liver) |
| Harries 85 | Article | 1985 | RIA | Automatized | home-made | Other |
| Hashida 90 | Article | 1990 | Fluorimetry | Single apparatus | home-made | Not reported |
| Haux 88 | Abstract | 1988 | Turbidimetry | Automatized | Boehringer Mannheim (704 system) | Not reported |
| Hendriks 00 | Article | 2000 | Chemiluminiscence | Automatized | Siemens (ACS Centaur), Abbott (Architect i2000), Roche (Elecsys 2010), Siemens (Immulite 2000), Johnson and Johnson (Vitros ECi) | Not reported |
| Hebert 78 | Abstract | 1978 | RIA | Single apparatus | home-made | Not reported |
| Herrera 92 | Abstract | 1992 | EIA | Automatized | Becton Dickinson affinity | Not reported |
| Hubl 05 | Article | 2005 | Chemiluminiscence | Automatized | Abbott (Architect ci8200) | Not reported |
| Iacobello 84 | Article | 1984 | RIA | Automatized | commercial | Not reported |
| Iacobello 86 | Abstract | 1986 | ELISA | Automatized | Bohering Mannheim (ES-22) | WHO STD reference material (liver) |
| Ihara 87 | Article | 1987 | EIA | Single apparatus | home-made | Not reported |
| Imagawa 82 | Article | 1982 | ELISA | Single apparatus | home-made | Not reported |
| ICSH 84 | Article | 1984 | Preparation of international standard of human ferritin |  |  | WHO STD reference material |
| ICSH 85 | Article | 1985 | Preparation of international standard of human ferritin |  |  | WHO STD reference material |
| Ishikawa 82 | Article | 1982 | ELISA | lab-equip | home-made | Not reported |
| Jennifer 84 | Abstract | 1984 | Chemiluminiscence | Automatized | DiaSorin (LIAISON XL) | Not reported |
| Jirinzu 88 | Abstract | 1988 | Fluorimetry | Automatized | Dade (stratus) | Not reported |
| Johnson 94 | Abstract | 1994 | EIA | Automatized | Microgenics Olympus (Cedia) | Not reported |
| Jones 75 | Article | 1975 | IRMA | Single apparatus | Searle (analmatic) | Not reported |
| Kahn 79 | Abstract | 1979 | IRMA | Single apparatus | Behring (Ria-gnost) | Not reported |
| Kamei 17 | Article | 2017 | Chemiluminescence | Automatized | 10 assay kits | WHO STD reference material |
| Kanamori 83 | Article | 1983 | RIA | Single apparatus | commercial | Not reported |
| Kano 2013 | Abstract | 2013 | Turbidimetry | Automatized | Hitachi 917 analyzer. | Not reported |
| Karakochuk 16 | Article | 2017 | ELISA, Chemiluminiscence, MEIA | Automatized | s-ELISA, AxSYM™ analyzer, Centaur® XP analyzer, Elecsys® 2010 | WHO STD reference material |
| Khosravi 88 | Article | 1988 | Fluorimetry | Single apparatus | home-made | WHO STD reference material recombinant |
| Kimura 13 | Abstract | 2013 | EIA | Single apparatus | ASKA (PSF−100), ASKA (PSF−500) | Other |
| Konig 99 | Article | 1999 | Chemiluminiscence | Automatized | Byk-Sangtec (Liason) | Not reported |
| Konijn 82 | Article | 1982 | Fluorimetry | Automatized | home-made | Not reported |
| Koseki 79 | Article | 1979 | IRMA | Single apparatus | Daichi (SPAC) | Not reported |
| Kotajima 03 | Article | 2003 | Turbidimetry | Single apparatus | Denka Seiken (Fer-Latex X2) | Other |
| Kousaka 84 | Article | 1984 | RIA | Single apparatus | commercial | Not reported |
| Kristensen 16 | Article | 2016 | ELISA, Turbidimetry, Chemiluminiscence | Automatized | Roche Cobas, Roche Modular, Abbott Architect, Beckman Coulter Unicel and Siemens ADVIA Centaur | WHO STD reference material |
| Kubasik 87 | Abstract | 1987 | EIA | Single apparatus | Beckman Coulter (epsilon) | Not reported |
| Kubo 78 | Article | 1978 | IRMA | Automatized | Behring (Ria-gnost) | Not reported |
| Kubota 14 | Abstract | 2014 | Turbidimetry | Automatized | N-assay LA Ferritin (NITTOBO, Tokyo, Japan) on TBA-40FR Accute (TOSHIBA, Tokyo, Japan) | Not reported |
| Kwiatkowski 84 | Abstract | 1984 | RIA | Single apparatus | Corning (Magic) | Not reported |
| Lavigne 92 | Abstract | 1992 | Fluorimetry | Automatized | PB Diagnostic Systems (OPUS) | WHO STD reference material (liver) |
| Lee 81 | Article | 1981 | EIA | Single apparatus | home-made | Not reported |
| Lee 85 | Article | 1985 | EIA | Single apparatus | Beckman (biotin-avidin) | Not reported |
| Lee 87 | Article | 1987 | ELISA, RIA | Single apparatus | home-made | Not reported |
| Letellier 96 | Article | 1996 | EIA | Automatized | Bayer (Technicon Immuno I) | Other |
| Lipschitz 81 | Article | 1981 | RIA, IRMA | Single apparatus | Clinical Assays (Gamma Dab), Ramco (Fer-Iron) | WHO STD reference material (spleen, liver and recombinant) |
| Lotz 99 | Article | 1999 | Turbidimetry, chemiluminiscence, Nephelometry, EIA, MEIA | Automatized | Roche (Tina-quant), Roche (Enzymun), Abbott (Imx), Behring (N-Latex), Bayer (ACS 180) | WHO STD reference material recombinant and other |
| Milman 94 | Abstract | 1994 | RIA | Automatized | Amersham (RIA) | WHO STD reference material (liver) |
| Milman 97 | Abstract | 1997 | MEIA | Automatized | Abbott (AxSYM) | WHO STD reference material (liver) |
| Mitsuma 84 | Article | 1984 | RIA | Single apparatus | commercial | Not reported |
| Molinaro 2015 | Article | 2015 | chemiluminiscence, Turbidimetry | Automatized | BioSystems (BA 400 LED Technology), Abbott (Architect i4000) | WHO STD reference material (spleen) |
| Murthy 95 | Article | 1995 | EIA | Automatized | Siemens (Technicon Immuno I) | Other |
| Ng 83 | Article | 1983 | IRMA, RIA, EIA | Automatized | Ramco (Fer-Iron), Corning (Immo phase I), Corning (Immo phase II), Clinical Assays (Gamma Dab), Abbott (Ferrizyme) | Not reported |
| Novembrino 05 | Article | 2005 | MEIA | Automatized | Abbott (Imx) | Not reported |
| Picelli 92 | Abstract | 1992 | Fluorimetry | Automatized | Biomerieux (VIDAS ferritin) | WHO STD reference material (spleen) |
| Pierson Perry 94 | Abstract | 1994 | EIA | Automatized | Dupont (Aca plus) | Not reported |
| Pocekay 92 | Abstract | 1992 | EIA | Automatized | Bio-Rad (RADIAS) | Not reported |
| Polson 88 | Article | 1988 | ELISA | Single apparatus | home-made | Not reported |
| Prior 85 | Abstract | 1985 | RIA, EIA | Automatized | Becton Dickinson (RIA), Abbott (Ferrizyme), Hybritech (Tandem-E) | Not reported |
| Ramakhrishnan 91 | Abstract | 1991 | Chemiluminiscence | Single apparatus | home-made | Not reported |
| Ramm 90 | Article | 1990 | ELISA | Single apparatus | home-made | WHO STD reference material (spleen) |
| Ranjitkar 16 | Article | 2016 | Chemiluminiscence | Automatized | commercial | WHO standard |
| Raymond 92 | Abstract | 1992 | Chemiluminiscence | Automatized | Bayer (ACS 180) | Not reported |
| Revenant 83 | Article | 1983 | ELISA | Single apparatus | home-made | Not reported |
| Revenant 85 | Abstract | 1985 | ELISA | Single apparatus | home-made | WHO STD reference material (liver) |
| Robinson 91 | Abstract | 1991 | RIA | Single apparatus | Not reported | Not reported |
| Rohner 05 | Article | 2005 | ELISA, Chemiluminiscence | Single apparatus | Ramco (Spectro Ferritin), Siemens (Immulite 1) | WHO STD reference material recombinant |
| Sampson 94 | Abstract | 1994 | MEIA, Chemiluminiscence, RIA | Automatized | Abbott (Imx), Siemens (Immulite 1), Sanofi (Access), Corning (Magic) | Not reported |
| Schneider 91 | Abstract | 1991 | EIA | Automatized | Becton Dickinson (affinity) | WHO STD reference material (liver) |
| Shimakawa 14 | Abstract | 2014 | Turbidimetry | Automatized | TBA-2000FR automated clinical chemistry analyzer (Toshiba). | Not reported |
| Shindelman 92 | Abstract | 1992 | EIA | Automatized | Microgenics Olympus (Cedia) | Not reported |
| Signo 05 | Article | 2005 | Turbidimetry | Automatized | Denka Seiken (Fer-Latex X2) | WHO STD reference material recombinant |
| Simo 94 | Article | 1994 | Turbidimetry | Automatized | Instrumentation Laboratories (Monarch 2000) | Other |
| Simondsen 93 | Abstract | 1993 | MEIA | Automatized | Abbott (AxSYM) | Not reported |
| Skikne 84 | Article | 1984 | IRMA | Automatized | Hybritech (IRMA mono), Hybritech (IRMA poli) | WHO STD reference material (liver) |
| Smith 94 | Abstract | 1994 | Chemiluminiscence | Automatized | Sanofi (Access) | Not reported |
| Spillane 98 | Article | 1998 | ELISA | Single apparatus | home-made | Not reported |
| Standefer 93 | Abstract | 1993 | LPIA | Automatized | Mitsubishi (LPIA-100) | Not reported |
| Steele 05 | Article | 2005 | Chemiluminiscence | Automatized | Bayer Advia Centaur Beckman Access/2 Dade Dimension HM DPC Immulite 2000 Roche Elecysys 1010/2010 Roche Tina-Quant Vitros ECi | Not reported |
| Theriault 77 | Article | 1977 | ELISA | Automatized | home-made | Not reported |
| Timmons 84 | Abstract | 1984 | Fluorimetry | Automatized | home-made | Not reported |
| Trefz 94 180. | Abstract | 1994 | Turbidimetry | Automatized | Roche (Unimate Latex) | Not reported |
| Van Oost 82 | Article | 1982 | RIA, IRMA | Single apparatus | University Utrecht (Utrecht-kit), Clinical Assays (Gamma Dab), Ramco (Fer-Iron), Hoechst (RIA) | Not reported |
| Vasileff 94 | Abstract | 1994 | MEIA | Automatized | Abbott (AxSYM) | Not reported |
| Velazquez 93 | Abstract | 1993 | Chemiluminiscence | Automatized | Boehringer Mannheim (Enzyme-Test CYFRA) | Not reported |
| Vernet 89 | Abstract | 1989 | Fluorimetry | Automatized | Baxter-Travenol (Stratus) | WHO STD reference material (liver) |
| Vernet 99 | Article | 1999 | Chemiluminiscence | Automatized | Johnson and Johnson (Vitros ECi) | WHO STD reference material (spleen) |
| Wang 09 | Article | 2009 | EIA | Single apparatus | home-made | Not reported |
| Watanabe 79 | Article | 1979 | ELISA | Single apparatus | home-made | Not reported |
| Wells 93 | Abstract | 1993 | Chemiluminiscence | Single apparatus | Siemens (Vista) | WHO STD reference material (liver) |
| Wide 77 | Article | 1977 | RIA | Single apparatus | home-made | Not reported |
| Wilding 93 | Abstract | 1993 | EIA | Single apparatus | Siemens (Technicon Immuno I) | Other |
| Wood 81 | Article | 1981 | 7 RIA vs 4 IRMA | Single apparatus | Clinical Assays Travenol (GammaDab RIAa, GammaDab RIAb), Behringwerke AG (D-gnost Ferritin), New England Nuclear (RIANEN), Ramco Lab (FER-IRON), Amersham (Ferritin RIA), Becton Dickinson (Ferritin RIA), Pharmacia (PRIST), DP Corporation (Ferritin RIA), Serono-Biodata (Ferritin Kit), Daiichi Radioisotopes (SPAC) | Other |
| Yajima 84 | Article | 1984 | IRMA | Single apparatus | home-made | Not reported |
| Yamamoto 92 | Abstract | 1992 | Chemiluminiscence | Single apparatus | Sankyo (Luminomaster) | Not reported |
| Zhang 99 | Article | 1999 | ELISA | Single apparatus | home-made | Not reported |
| Zhang 15 | Article | 2015 | Chemiluminiscence | Automatized | Architect i2000 system Abbott Laboratories and Cobas E601Roche Diagnostics. | Not reported |
| Zhang 06 | Article | 2006 | EIA | Single apparatus | home-made | Not reported |

^1^ EIA: Enzyme immunoassay; ELISA: Enzyme linked immunosorbent assay; MEIA: microparticle enzyme immunoassay; RIA: Radioimmune assay; IRMA: Immunoradiometric assay; LPIA Latex photometric immunoassay; RPIA: Radial partition immunoassay
