# Supplementary material for: Performance and comparability of laboratory methods for measuring ferritin concentrations in human serum or plasma: A systematic review and meta-analysis
Source: PLoS One. 2018 May 3;13(5):e0196576. doi: 10.1371/journal.pone.0196576 (PMC5933730; doi:10.1371/journal.pone.0196576)
Supplement: S2 Table — (DOCX) [file pone.0196576.s002.docx]

**Supporting information**

S2 Table. Summary of studies reporting Bland-Altman (B-A) statistics: mean and standard deviation of the regression intercept and slope between different detection techniques.

| **Study ID** | **Method subtype compared^1^** | **B-A's differences Mean (µ)** | **B-A's corresponding SD (σ)** | **Lower limit of agreement (µ-2σ)** | **Higher limit of agreement (µ+2σ)** | **n** |
| --- | --- | --- | --- | --- | --- | --- |
| **Karakochuk 2017^115^** | ELISA vs MEIA^2^ | 44 | 27.1 | -9.2 | 97.2 | 407 |
| **Karakochuk 2017^115^** | ELISA vs MEIA^3^ | 43.2 | 27.5 | -10.7 | 97.1 | 100 |
| **Karakochuk 2017^115^** | ELISA vs CHEM^4^ | 34.6 | 25.6 | -15.58 | 84.8 | 100 |
| **Karakochuk 2017^115^** | MEIA vs CHEM | -8.6 | 12.8 | -33.7 | 16.5 | 100 |
| **Karakochuk 2017^115^** | ELISA vs CHEM^5^ | -11.5 | 29.8 | -69.9 | 46.9 | 209 |
| **Dipalo 2016^62^** | CHEM vs CHEM | 34 | 12.2 | 10 | 58 | 95 |
| **Molinario 2015^142^** | TURB vs CHEM | -7.9 | 1.5 | -10.9 | -4.84 | 111 |
| **Zhang 2015^198^** | CHEM vs CHEM | 60.6 | 70.9 | -78.3 | 199.5 | 125 |
| **Rohner 2005^162^** | ELISA vs CHEM | -8.0 | 8.5 | -25.1 | 9.1 | 51 |
| **Erhardt 2004^68^** | ELISA vs IRMA | 3.7 | 12.6 | -21.5 | 28.9 | 41 |
| **Gomez 2000^79^** | TURB vs CHEM | -6.0 | 8.0 | -22.0 | 10.0 | 100 |

^1^ ELISA: Enzyme linked immunosorbent assay; MEIA: microparticle enzyme immunoassay; IRMA: Immunoradiometric assay; TURB: turbidimetric assay; CHEM: chemiluminescent assay.
