# Supplementary material for: Performance and comparability of laboratory methods for measuring ferritin concentrations in human serum or plasma: A systematic review and meta-analysis
Source: PLoS One. 2018 May 3;13(5):e0196576. doi: 10.1371/journal.pone.0196576 (PMC5933730; doi:10.1371/journal.pone.0196576)
Supplement: S3 Table — (DOCX) [file pone.0196576.s003.docx]

**Supporting information**

S3 Table. Laboratory assessment data from a quality control program of the Spanish Society of Clinical Biochemistry and Molecular Pathology (2014)

| Automated equipment | Mean | CV^1^ (%) | SD^2^ | 95% confidence interval | | Number of labs |
| --- | --- | --- | --- | --- | --- | --- |
|  |  |  |  | Lower limit | Upper limit |  |
| Cobas 6000 (e601), Cobas 8000 (e602), Modular Analytics E-170 | 38.00 | 9.84 | 3.74 | 36.74 | 39.26 | 34 |
| Cobas c701, Cobas c702 | 43.60 | 8.29 | 3.61 | 42.35 | 44.85 | 32 |
| Cobas c501, Cobas c502, cobas c311 | 39.60 | 7.52 | 2.98 | 38.43 | 40.77 | 25 |
| Architect series I | 32.00 | 9.56 | 3.06 | 30.80 | 33.20 | 25 |
| Centaur, Centaur XP | 31.10 | 11.50 | 3.58 | 29.64 | 32.56 | 23 |
| Beckman Coulter AU | 34.70 | 13.10 | 4.55 | 32.66 | 36.74 | 19 |
| Advia | 34.60 | 9.25 | 3.20 | 33.16 | 36.04 | 19 |
| Dimension Vista | 29.40 | 9.29 | 2.73 | 27.79 | 31.01 | 11 |

^1^Coefficient of variation

^2^Standard deviation
