# Supplementary material for: Performance and comparability of laboratory methods for measuring ferritin concentrations in human serum or plasma: A systematic review and meta-analysis
Source: PLoS One. 2018 May 3;13(5):e0196576. doi: 10.1371/journal.pone.0196576 (PMC5933730; doi:10.1371/journal.pone.0196576)
Supplement: S2 File — (PDF) [file pone.0196576.s005.pdf]

UNIVERSITY of York
Centre for Reviews and Dissemination

NHS
National Institute for Health Research

PROSPERO International prospective register of systematic reviews

Review title and timescale

1 **Review title**
Give the working title of the review. This must be in English. Ideally it should state succinctly the interventions or exposures being reviewed and the associated health or social problem being addressed in the review.
Accuracy and comparability of methods for measuring ferritin concentration to determine iron deficiency, repletion and overload

2 **Original language title**
For reviews in languages other than English, this field should be used to enter the title in the language of the review. This will be displayed together with the English language title.

3 **Anticipated or actual start date**
Give the date when the systematic review commenced, or is expected to commence.
06/10/2015

4 **Anticipated completion date**
Give the date by which the review is expected to be completed.
01/06/2016

5 **Stage of review at time of this submission**
Indicate the stage of progress of the review by ticking the relevant boxes. Reviews that have progressed beyond the point of completing data extraction at the time of initial registration are not eligible for inclusion in PROSPERO. This field should be updated when any amendments are made to a published record.

The review has not yet started ✖

Review stage	Started	Completed
Preliminary searches	No	Yes
Piloting of the study selection process	Yes	No
Formal screening of search results against eligibility criteria	Yes	No
Data extraction	No	No
Risk of bias (quality) assessment	No	No
Data analysis	No	No

Provide any other relevant information about the stage of the review here.

Review team details

6 **Named contact**
The named contact acts as the guarantor for the accuracy of the information presented in the register record.
Maria Nieves Garcia-Casal

7 **Named contact email**
Enter the electronic mail address of the named contact.
mngarciacasal@gmail.com

8 **Named contact address**
Enter the full postal address for the named contact.
World Health Organization. Department of Nutrition for Health and Development Evidence and Programme Guidance Unit 20 Av Appia Geneva, Switzerland

9 **Named contact phone number**
Enter the telephone number for the named contact, including international dialing code.
+41227911893

10 **Organisational affiliation of the review**
Full title of the organisational affiliations for this review, and website address if available. This field may be completed as 'None' if the review is not affiliated to any organisation.
World Health Organization
Website address:
who.int

11 **Review team members and their organisational affiliations**
Give the title, first name and last name of all members of the team working directly on the review. Give the organisational affiliations of each member of the review team.

Title	First name	Last name	Affiliation
Dr	Maria Nieves	Garcia-Casal	Evidence and Programme Guidance, Department of Nutrition for Health and Development, World Health Organization, Geneva, Switzerland
Dr	Juan Pablo	Pena-Rosas	Evidence and Programme Guidance, Department of

			Nutrition for Health and Development, World Health Organization, Geneva, Switzerland
Dr	Eloisa	Urrechaga	Hospital Galdakao Usansolo, Galdakao, Spain
Dr	Jesus Fernando	Escanero	Department of Pharmacology and Physiology, Faculty of Medicine, University of Zaragoza, Zaragoza, Spain
Dr	Junsheng	Huo	Department of Food Science and Technology, Institute of Nutrition and Food Safety, Beijing, China
Dr	Ricardo X	Martinez	World Health Organization, Hamburg, Germany
Dr	Lucero	Lopez-Perez	World Health Organization, Vence, France

12 Funding sources/sponsors

Give details of the individuals, organizations, groups or other legal entities who take responsibility for initiating, managing, sponsoring and/or financing the review. Any unique identification numbers assigned to the review by the individuals or bodies listed should be included.

The review is mthe responsability of hte Evidence and Programme Guidance Unit, Department of Nutrition for Health and Development at the World Health Organization and receives financial support from International Micronutrient Malnutrition Prevention and Control (IMMPaCt) Program, Division of Nutrition, Physical Activity and Obesity at the US Centers for Disease Control and Prevention (CDC) (USA), United States Agency for International Development (USAID) (USA), and the Bill & Melinda Gates Foundation (USA)

13 Conflicts of interest

List any conditions that could lead to actual or perceived undue influence on judgements concerning the main topic investigated in the review.

Are there any actual or potential conflicts of interest?

None known

14 Collaborators

Give the name, affiliation and role of any individuals or organisations who are working on the review but who are not listed as review team members.

Title	First name	Last name	Organisation details

Review methods

15 Review question(s)

State the question(s) to be addressed / review objectives. Please complete a separate box for each question.

To compare the accuracy and comparability of the most common methods used for serum, plasma and erythrocyte ferritin concentration determinations to detect iron deficiency, repletion or overload.

Secondary objective To determine the sensitivity, specificity and predictive value between ferritin methods to assess iron status in general population.

Secondary objective To assess the variability of serum, plasma and erythrocyte ferritin concentrations using different laboratory methods of detection, and estimate inter-methods adjustments.

Secondary objective To review the use of international standard materials of ferritin for calibration purposes and in global public health surveillance.

16 Searches

Give details of the sources to be searched, and any restrictions (e.g. language or publication period). The full search strategy is not required, but may be supplied as a link or attachment.

Electronic searches We will search the following international and regional databases for all available years. 1. Cochrane Central Register of Controlled Trials (CENTRAL; part of The Cochrane Library, which includes the specialised register of the Cochrane Developmental, Psychosocial and Learning Problems Group). 2. MEDLINE (OVID). 3. Ovid Medline (R) In-process, and other Non-indexed Citations. 4. Embase (OVID). 5. CINAHL (EBSCOhost) 6. Science Citation Index (Web of Science). 7. Conference Proceedings Citation Index-Science (Web of Science). 8. BIOSIS Previews (Web of Science). 9. Database of Abstracts of Reviewsof Effects (DARE; part of The Cochrane LIbrary). 10. Cochrane Database of Systematic Review (CDSR; part of The Cochrane LIbrary). 11. ARIF Reviews Database (birmingham.ac.uk/research/activity/mds/projects/HaPS/PHEB/ARIF/databases/index.aspx). 12. PROSPERO (crd.york.ac.uk/PROSPERO). 13. IBECS (ibecs.isciii.es). 14. Scielo (scielo.br/). 15. Global Index Medicus - AFRO (includes African Index Medicus); 16. EMRO (includes Index Medicus for the Eastern Mediterranean Region). 17. LILACS (lilacs.bvsalud.org/en). 18. PAHO (Pan American Health Library). 19. WPRO (includes Western Pacific Region Index Medicus). 20. IMSEAR, Index Medicus for the South-East Asian Region. 21. IndMED, Indian medical journals (indmed.nic.in). 22. Proquest Dissertations and Theses. Searching other resources For assistance in identifying ongoing or unpublished studies, we will contact the Department of Nutrition for Health and Development and the regional offices of the WHO, as well as the nutrition section of the Centers for Disease Control and Prevention (CDC), National Institutes of Health (NIH), ILSI Europe and WHO collaborating centres. We will contact authors of included studies and check reference lists of included papers and relevant reviews for identification of additional records. We will not apply language or publication date restrictions for any database. If we identify articles written in a language other than English, we will commission their translations into English.

17 URL to search strategy

If you have one, give the link to your search strategy here. Alternatively you can e-mail this to PROSPERO and we will store and link to it.

18 Condition or domain being studied

Give a short description of the disease, condition or healthcare domain being studied. This could include health and

wellbeing outcomes.

Ferritin is an iron storage protein present in all cells of the organism. A small amount is found in plasma and serum, which is a reflection of iron stores in normal individuals. While a low serum ferritin is usually regarded as an indicator of iron depletion, the result must be interpreted with caution in any patient with an underlying inflammatory process, as ferritin is an acute phase reactant, and is increased in case of acute or chronic inflammatory processes. Virtually all patients with low serum iron and low serum ferritin have iron deficiency. Patients may have a low or normal serum iron level and normal haemoglobin in the presence of decreased ferritin, indicating an iron-depletion state before anaemia develops. Ferritin may be useful in helping distinguish between iron-deficiency anaemia and functional iron deficiency (anaemia of inflammation). Since ferritin concentration is widely used as marker of iron stores and status, it is important to determine if all methods commonly used to assess ferritin concentrations are capable of detecting and discriminating all possible iron statuses (deficiency, repletion, and overload), and to evaluate if all methods are comparable. An ongoing Cochrane review assessing serum or plasma ferritin concentration as an index of iron deficiency and overload is being conducted as part of the evidence retrieval, summary, and assessment to inform recommendations on the use of ferritin as a marker of iron status in populations. It seeks to evaluate ferritin concentration as an index of iron deficiency (where iron deficiency is defined by no stainable iron in bone marrow biopsies) or iron overload (where iron overload is defined by liver biopsies data), and to assess the quantity and quality of evidence available to support the selection of thresholds of ferritin that define iron deficiency and iron overload. The present review will compare methods for detecting ferritin, with emphasis on performance and comparability. There are various tests to determine serum, plasma or erythrocyte ferritin concentration. The vast majority of them are based in antigen-antibody reactions that are detected by different means. The detection could be by radioactive counting or by colour development using different enzyme-substrate pairs to measure colour development at visible range. Also, light scattering or turbidimetric measurements have been developed as well as fluorescence emission. The first reported methods for ferritin determination were radioactive, using radioisotopes to label the antigen (IRMA (immunoradiometric assay)) or the antibody (RIA (radioimmunoassay)). Common colorimetric methods include peroxidase or glycosidase based colour development. Methods based in light passage could be turbidimetric or nephelometric. Another difference in the development of methods for ferritin determination has been automatisation. Radioactive methods started as home made in a few laboratories, followed in few years by colorimetric tests. Both methodologies were improved and commercialised, making them widely available. More recently, automated equipments that allow the determination of various metabolites, including ferritin, have been developed. The detection method for this equipment varies, but is mainly based on turbidimetry and chemiluminescence. The performance of some of the methods to determine ferritin has been compared over time. The analysis of performance between variants of immuno enzymatic ferritin determinations, such as displacement radioimmunoassay, enzyme immunoassay, and immunoradiometric assay, showed that these methods did not compare well. On the other hand, the comparison of radioimmunoassay with immuno turbidimetric assay and chemiluminescence detection for determination of ferritin in serum samples showed that both are good alternatives to substitute isotopic methods. Turbidimetric methods have also been reported to correlate well with data obtained by nephelometry and radioimmunoassay (RIA).

19 Participants/population

Give summary criteria for the participants or populations being studied by the review. The preferred format includes details of both inclusion and exclusion criteria.

We will include studies reporting ferritin concentrations from human population samples (serum, plasma or erythrocytes) without age or gender restrictions that are apparently healthy, iron deficient and/or iron overload. We will also include studies from infection/inflammation settings, malaria areas, disaster or emergency areas where respiratory and gastrointestinal infections are common, bacterial versus viral infections, acute versus chronic conditions, and data from diabetic, obese, overweight and/or, insulin-resistant individuals. The origin of inflammation (infection, chronic disease, non-infectious disease) will be a sub classification criterion.

20 Intervention(s), exposure(s)

Give full and clear descriptions of the nature of the interventions or the exposures to be reviewed

Measurement of iron status in the general population is important to determine the prevalence and distribution of iron deficiency and overload, and thus to decide appropriate interventions, and to monitor and evaluate the impact and safety of implemented public health programmes. The selection of a test that reflects real ferritin concentration and has been validated through the use of reference materials has important implications from the perspective of the individual and public health. The comparability of results from patients to diagnose iron deficiency or determine risk for iron overload has important implications for clinical decisions and also the appropriate use of resources. It is important to determine whether a therapeutic decision and treatment is having the expected effect and is not causing harm, regardless of the method used. Likewise, in the planning and evaluation of public health interventions it is important to determine the iron status of the targeted population as well as the impact of a nutrition intervention. If different laboratory methods give different results, this may lead to misinterpretation of the effects and lead to incorrect public health decisions. Additionally, it should be possible to compare data from different surveys performed years apart and performed by different methods. The use of different laboratory methods to assess ferritin concentrations (homemade, commercial or automated) cannot be comparable unless they are assessed and calibrated against an established reference standard. Also, determining the use of this material at laboratory level or in surveys could be of use in global public health surveillance.

21 Comparator(s)/control

Where relevant, give details of the alternatives against which the main subject/topic of the review will be compared (e.g. another intervention or a non-exposed control group).

There are different tests used in the determination of ferritin to diagnose iron deficiency or risk of iron overload. To evaluate the accuracy to detect iron deficiency or risk of iron overload using different ferritin methods, we will use the

assessment of iron deficiency/overload using radiometric and colorimetric assays. Measurement of ferritin can be performed with homemade, commercial or automated methods requiring separation of serum or plasma from the cellular component of whole blood. There are several detection platforms for quantifying ferritin and the global availability of these assays and equipments may lead to variations between studies and to difficulties when addressing the comparability between methods used.

22 Types of study to be included initially

Give details of the study designs to be included in the review. If there are no restrictions on the types of study design eligible for inclusion, this should be stated.

For all proposed objectives, information will be retrieved from published literature (prospective, retrospective, sectional, longitudinal and case-control studies) containing the characteristics and performance of at least two methods for ferritin determination for human use, reporting the detailed procedures and performance parameters. We will also include studies evaluating the performance of two or more ferritin methods comparing the same human samples or using international reference materials. We will include studies reporting different methods for ferritin determinations when testing human serum, plasma or erythrocytes samples, and when comparing homemade, commercial or automated methods to determine: principle of method, calibration to international reference material, limit of detection, reference interval, linearity, interferences and cost. Studies and manufacturers reports describing and validating methods using international reference materials for ferritin from human sources or recombinant origin.

23 Context

Give summary details of the setting and other relevant characteristics which help define the inclusion or exclusion criteria.

Index tests. Serum/plasma/erythrocytic ferritin concentration detected by any available test (radiometric, colorimetric, turbidimetric, luminescent assays), performed manually or automatically, and from homemade, commercial or automated equipment origin. Target conditions. Iron deficiency, iron repletion, and iron overload as defined by manufacturer, trialists or World Health Organisation (WHO) cutoff points. Reference standards. Although there is no method that can be considered the reference standard for ferritin determinations, comparisons will be made against radiometric (RIA) and colorimetric (ELISA) methods, the most commonly used methods for ferritin determinations. In order to asses iron status, we will use the existing cut-off points for serum ferritin that were summarized by WHO in 2011 as a measure of iron stores. For depleted iron stores the cut-offs are < 12 µg/L for children less than five years of age and < 15 µg/L for other age groups. If infection is present the cut-off is < 30 µg/L for children less than five years of age, without data for other age groups. For severe risk of iron overload in adults the cutoff is 200 mg/L. There is no difference in cut-off points for gender, although data in a non-iron deficient population indicates that in late childhood and adolescence males have higher ferritin concentrations than females. This pattern persists up to adulthood and after menopause, when ferritin concentrations increase. International reference materials. The use of the international reference materials to develop tests or to evaluate inter-laboratories performance is desirable. International reference materials for ferritin have been developed for calibrating working standards in the routine ongoing assays performed in laboratories and also for evaluating and standardizing new tests for ferritin quantification. The World Health Organization (WHO) Expert Committee on Biological Standardization established 94/572 as the third international reference material for ferritin. This international standard for ferritin is commercially available worldwide.

24 Primary outcome(s)

Give the most important outcomes.

Comparisons between at least 2 methods for ferritin determinations detailing: sensitivity, precision, accuracy, predictive values, inter-methods adjustment, use of international reference materials

Give information on timing and effect measures, as appropriate.

25 Secondary outcomes

List any additional outcomes that will be addressed. If there are no secondary outcomes enter None.

Quality assurance data, cost

Give information on timing and effect measures, as appropriate.

26 Data extraction, (selection and coding)

Give the procedure for selecting studies for the review and extracting data, including the number of researchers involved and how discrepancies will be resolved. List the data to be extracted.

The data retrieved in each search will be screened independently by two review authors (MNGC, EU) to assess eligibility as determined by the comparisons and criteria listed above. When a title or abstract cannot be rejected with certainty, we will obtain the full text of the article for further evaluation. We will retrieve full copies of all eligible papers. If full articles cannot be obtained, we will attempt to contact the authors to obtain further details of the study. Failing this, studies will be classified as "awaiting assessment" until further information is published or made available to us. Disagreements at any stage of the eligibility assessment process will be resolved through discussion and consultation with a third author (JPPR), where necessary. Data will be independently extracted by two review authors (JH and JFE), using data extraction forms tested and approved by all review authors in order to enhance consistency amongst reviewers. The form will be modified if necessary. We will collect information on study design, study setting, participants (number and characteristics), and methods of ferritin quantification, and will provide a full description of the methods used. Details of outcomes measured (including a description of how, when, and the methods by which outcomes were measured) and results will be extracted. When information regarding any aspect of study design or results is unclear, we will attempt to contact authors of the original reports, asking them to provide further details.

27 Risk of bias (quality) assessment

State whether and how risk of bias will be assessed, how the quality of individual studies will be assessed, and whether and how this will influence the planned synthesis.

We will assess risk of bias and applicability using the quality assessment of diagnostic accuracy studies (QUADAS-2)

tool. We will rate each of the four key domains (patient selection, index test, reference standard, flow and timing) using the signalling questions as developed by the QUADAS-2 group. Two review authors (MNGC, JPPR) will independently assess the included studies and will resolve any disagreement by discussion or by involving an additional review team member (EU). We will score all items in the QUADAS-2 tool as ‘yes’, ‘no’ or ‘unclear’, except for risk of bias and concerns regarding applicability, which will be scored as high/low/unclear. We will use graphs to present overall scores of risk of bias and applicability for each domain.

28 Strategy for data synthesis

Give the planned general approach to be used, for example whether the data to be used will be aggregate or at the level of individual participants, and whether a quantitative or narrative (descriptive) synthesis is planned. Where appropriate a brief outline of analytic approach should be given.

In order to compare the accuracy of the assessment of iron deficiency/overload using different ferritin measurement methods, we will use as reference standard the assessment of Iron deficiency/overload using radiometric (RIA) and colorimetric (ELISA) assays. Most of the available studies compare ferritin measures using different methods, but do not directly assess Iron deficiency/overload. In order to obtain an index test result we will apply the WHO recommended threshold test to the set of ferritin measures obtained with the index method and consider as our reference test result the outcome of the same threshold on the ferritin measures made using the radiometric, colorimetric, and turbidimetric assays. We will then compute the 2 x 2 table with true positives, false positives, false negatives, and true negatives from these outcomes. From these tables, and since we will be using a unique threshold, we will use the bivariate model to estimate the accuracy of the test and corresponding 95% confidence intervals. As complementary analyses, comparisons will be for the most used methods against each other to compare performance of methods, including indicators as sensitivity, precision (inter- and intra-assay coefficient of variation), accuracy, and others as mentioned below. We will use present coupled forest plots to depict sensitivity and specificity for each ferritin test method for iron deficiency and overload. For specific objective 2, we will use the following four-step statistical methodology will be used to compare the different laboratory methods/kits/procedures in the case of comparative indicators. 1. Re-group all couples of methods/kits/procedures within the study as well as between all selected studies. Thus, we will have couples of methods/kits/procedures to compare such as ELISA versus radiometric, EIA (enzyme immunoassay) versus radiometric. 2. Provide either fixed-effect or random-effects pooled estimates for the couple of methods concerning each of the three comparative indicators. 3. Do forest plots, funnel plots, sensitivity analysis, and investigations of heterogeneity for each of the indicators for couples of methods/kits/procedures. 4. Present summary tables with the results for all couples of methods/kits/procedures and comparative indicators. Concerning our third secondary objective (review the use of international reference materials), we will perform comparative tables for the most common used methods against each other (including homemade kits, commercial kits and automated equipments) for the following parameters: calibration to international reference materials, principle of method, limit of detection, reference interval, linearity, interferences and cost.

29 Analysis of subgroups or subsets

Give any planned exploration of subgroups or subsets within the review. ‘None planned’ is a valid response if no subgroup analyses are planned.

We will use covariate analysis, and develop SROC curves and accuracy estimates for the following groups (providing that there are enough studies available). 1. Ferritin test method: radiometric, colorimetric, turbidimetric, other. 2. Machine generation: first (1975 to 1990), second (1990 to 2000), third (2005 to date). 3. Blood derivatives from which the tests sample ferritin: serum, plasma or erythrocytes. 4. Development of assay: homemade, commercial, automated equipment. 5. Age: infants (less than one year of age), children (two to 11.9 years of age), adolescents (12 to 18.9 years of age), adults (19 years of age or older); 6. Gender: male, female, mixed/unreported. 7. Vulnerability to iron deficiency: infants, women of reproductive age, pregnancy. 8. Pregnancy status: pregnant versus non-pregnant versus mixed/unreported. 9. Body Mass Index (BMI): low weight (30 kg/m²). 10. Inflammation/infection: infected versus non-infected versus mixed/unknown/unreported and sub-classified by etiology and duration. 11. Performance: variability, reproducibility, and costs. We will limit this analysis to those covariates for which at least three studies contributed data. We will examine differences between covariates by visual inspection; non-overlapping confidence intervals (CIs) suggest a statistically significant difference in treatment effect between the subsets. We will also formally investigate differences between two or more covariate subsets (Borenstein 2009). We will conduct a covariate analysis using SAS.

Review general information

30 Type of review

Select the type of review from the drop down list.

Diagnostic

Methods performance

31 Language

Select the language(s) in which the review is being written and will be made available, from the drop down list. Use the control key to select more than one language.

English

Will a summary/abstract be made available in English?

Yes

32 Country

Select the country in which the review is being carried out from the drop down list. For multi-national collaborations select all the countries involved. Use the control key to select more than one country.

China, France, Germany, Spain, Switzerland

33 Other registration details

Give the name of any organisation where the systematic review title or protocol is registered together with any unique identification number assigned. If extracted data will be stored and made available through a repository such as the Systematic Review Data Repository (SRDR), details and a link should be included here.

34 Reference and/or URL for published protocol
Give the citation for the published protocol, if there is one.
Give the link to the published protocol, if there is one. This may be to an external site or to a protocol deposited with CRD in pdf format.

35 Dissemination plans
Give brief details of plans for communicating essential messages from the review to the appropriate audiences.
This review will be part of the process of retrieving, summarizing and assessing the evidence to inform WHO recommendations on the use and interpretation of ferritin concentrations for assessing iron status in populations.
Do you intend to publish the review on completion?
Yes

36 Keywords
Give words or phrases that best describe the review. (One word per box, create a new box for each term)
Ferritin methods
Serum/plasma/erythrocyte
Iron deficiency, repletion and overload
Accuracy
Comparability
International reference materials

37 Details of any existing review of the same topic by the same authors
Give details of earlier versions of the systematic review if an update of an existing review is being registered, including full bibliographic reference if possible.

38 Current review status
Review status should be updated when the review is completed and when it is published.
Ongoing

39 Any additional information
Provide any further information the review team consider relevant to the registration of the review.

40 Details of final report/publication(s)
This field should be left empty until details of the completed review are available.
Give the full citation for the final report or publication of the systematic review.
Give the URL where available.
